# Outlines of Modern Chemistry

**Published:** 1878-01

**Authors:** 


*Outlines of Modern Chemistry, Organic, based in part upon Riches' Manuel de Chimie.* By C. GILBERT WHEELER, Professor of Chemistry in the University of Chicago. New York and Chicago: A. S. Barnes & Co., 1877. pp. 231. Price, $1.75.

The want of a small and inexpensive text-book upon Organic Chemistry has long been felt by the teacher and student of this extensive department of science. This small work is designed to furnish an aid to those who have previously acquired a knowledge of inorganic Chemistry, and who have no opportunity to consult the larger works of Fownes, Roscoe and others. It was prepared with special reference to the wants of the medical student.

The typographical execution is, with the exception of one grave fault, of a class rarely equaled in science publications. We can excuse proof errors in periodic literature, but in a scientific work depending upon formulæ and equations for its value we expect exactness. We find under the chapter upon Organic Acids, in the first ten pages, five mistakes in the formulæ given.

Such a mistake as the following can not be excused. Formic Acid, $CH_3O_3 = \left.\begin{matrix} CH_2O \\ H \end{matrix}\right\} O$. Neither member of the equation is correct, the formula for Formic Acid being $CH_2O_2$. With a few similar corrections the book would be as near perfection as we could desire. We know of no other that can equal it as an elementary text book. C.

